# Unreeling the chromatin thread: a genomic perspective on organization around the periphery of the *Arabidopsis* nucleus

**DOI:** 10.1186/s13059-017-1239-6

**Published:** 2017-05-23

**Authors:** Fredy Barneche, Celia Baroux

**Affiliations:** 1 IBENS, Département de Biologie, Ecole Normale Supérieure, CNRS, Inserm, PSL Research University, F-75005 Paris, France; 2 Department of Plant and Microbial Biology, Zürich-Basel Plant Science Center, University of Zurich, Zollikerstrasse 107, 8008 Zürich, Switzerland

## Abstract

The first genome-wide examination of the chromatin landscape at the periphery of the plant cell nucleus reveals substantial enrichment of heterochromatin and *Polycomb*-based repressive chromatin.

The cell nucleus is a highly structured subcellular organelle that functionally houses the genome. The double-membrane nuclear envelope provides a physical interface that contributes to the compartmentalization of chromosomal domains that have distinct chromatin states and activities within the nuclear space. Our knowledge of chromatin organization and function in plant systems is rapidly expanding, and the roles of different nuclear peripheral components in structuring chromatin are also beginning to emerge [1]. In metazoans, a complex meshwork of intermediate filament proteins constitutes the so-called lamina at the inner nuclear membrane to which chromatin regions and associated factors are anchored [2]. Plants lack clear orthologs of lamin proteins. Instead, plant-specific components may serve as a lamina-like matrix, whose influence on chromatin organization and activity remains to be determined (reviewed in [3, 4]). In a recent study, Bi and colleagues identified genomic regions that are associated with the *Arabidopsis* NUCLEOPORIN1 (NUP1) protein, an inner subunit of the nuclear pore complex (NPC) [5] responsible for mRNA export that protrudes out into the lamina-like matrix (Fig. 1). This work provides a first comprehensive view of the chromatin landscape at the nuclear periphery in plants.

**Fig. 1:**
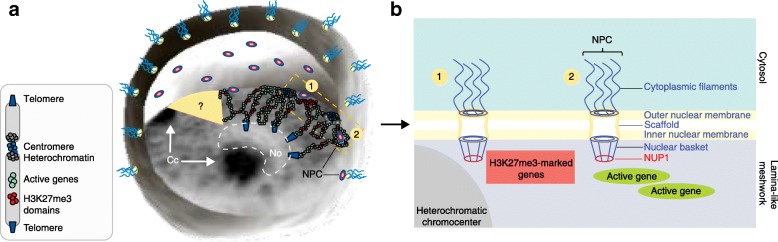
Chromatin landscape at the inner periphery of *Arabidopsis* nuclei. **a** Artist impression representing *Arabidopsis* interphase nuclei and the potential links between nuclear pore complexes (*NPC*) and contrasting chromatin contexts. Two NPCs neighboring (1) heterochromatic and *Polycomb*-repressed domains or (2) actively transcribed genes are highlighted within the yellow box. For simplicity, only two chromosomes are shown. Their telomeres are centrally localized, forming nucleolus-associated domains (NADs), whereas their heterochromatic repetitive elements are condensed around centromeric and peri-centromeric regions, forming peripheral chromocenters. According to the rosette organizational model described by Fransz et al. [6], gene-rich euchromatic loops emanate from the chromocenters. A question mark indicates the potential existence of gradually enriched RNA polymerase II transcription microenvironments from the nuclear interior to peripheral regions where mRNA surveillance and export could be favored. **b** Schematic representation of NUP1-containing NPCs facing diverse chromatin landscapes. NUP1 is densely distributed along the nuclear periphery and protrudes into the lamina-like nuclear matrix. The two NPCs boxed in **a** are schematically represented. *Cc* chromocenter, *No* nucleolus

## Functional interplay between nuclear components and genome topology

In *Arabidopsis* cells, most heterochromatin typically aggregates around the centromeric and peri-centromeric regions, forming conspicuous subnuclear foci referred to as chromocenters which are thought to tether transposable elements (TEs) from distant genomic domains [6] (Fig. 1a). With the exception of those containing 45S ribosomal DNA units and neighboring the nucleolus, *Arabidopsis* chromocenters are usually located near the nuclear periphery [6] and their establishment requires several lamina-like matrix components [3]. This hints at the presence of transcriptionally repressive environments in the vicinity of the plant nuclear envelope, like those found in budding yeast, nematodes, flies, and mammals. Notwithstanding this simple picture, reality might be more complex as the nuclear periphery also accommodates transcriptionally permissive environments. For example, artificial tethering of a transgene at the *Arabidopsis* NPCs enhanced its expression [7]. This positive link echoes the observation that endogenous *CAB* (*CHLOROPHYLL A/B-BINDING PROTEINS*) genes are repositioned from the interior to the periphery of *Arabidopsis* cotyledon nuclei when induced by light signaling [8]. Overall, the influence of peripheral proximity on gene expression remains poorly explored in plants [4], and is also the subject of intense investigations in metazoan cells, in which opposite trends have seemingly been observed in studies using differing approaches.

The integration of genome-wide ChIP-seq and Chromosome Conformation Capture (3C) approaches has recently extended linear epigenomic information on successive chromatin states towards a three-dimensional model of intra-chromosomal associations in *Arabidopsis*. Besides heterochromatic domains consisting mainly of highly condensed and silent elements, recent studies have revealed that genes typically display two major spatial chromatin configurations according to their transcriptional and epigenetic status: transcribed units fold into short chromatin loops, whereas genes marked by H3K27me3 and silenced by the Polycomb Repressive Complex 2 (PRC2) are frequently involved in long-range interactions between distant loci in plants [9] and mammals [1]. *Arabidopsis* chromosomes also form nucleolus-associated domains (NADs) that are enriched in transcribed ribosomal DNA genes and telomeric regions. Interestingly, NADs also contain heterochromatic TEs and silent protein-coding genes that might be sequestered in the nucleolus away from the RNA Pol II machinery (reviewed in [3]). Hence, increasing our knowledge of the functional significance of chromatin compartmentalization in plant species should shed light on evolutionarily conserved and divergent features of the control of genome expression in plant and animal cells.

## Plant NPCs interface a large fraction of the genome

The study by Bi and colleagues [5] offers a novel genomic perspective on chromatin subnuclear organization in plants. The authors used a restriction enzyme-ChIP (RE-ChIP) methodology to immunoprecipitate 7- to 12-kb-long chromatin fragments with NUP1, thus enriching for chromatin located preferentially at the nuclear periphery. Using this innovative approach, they found sequences covering 10–20% of the genome, with half of the identified genes (~3000) being commonly enriched with NUP1 in root and leaf tissues. This finding indicated robust association of a large gene set with the periphery across different somatic cell types, still implying substantial variations in chromatin organization between each sample type. For example, half of the NUP1-associated domains were not found in flowers, suggesting differences in genome topology between the cells of vegetative and reproductive tissues. These differences might relate to extensive gene expression reprogramming and large-scale chromatin reorganization events occurring during plant developmental transitions and plant cell differentiation [9].

## *Arabidopsis* NPCs are largely surrounded by transcriptionally repressed chromatin

Bi and colleagues also report that NUP1 RE-CHIP pulled down chromatin fragments that were enriched in acetylated histone H3 and other chromatin modifications characteristic of transcribed genes. The proximity between transcribed genes and NPCs might facilitate mRNA export via the so-called 'gene-gating process' proposed both for animal cells [2] and for the *Arabidopsis CAB* genes [3]. More surprisingly, Bi and colleagues further report that the NUP1-GFP chromatin landscape comprises many heterochromatic elements, in particular hundreds of long TEs that are silenced via a CHROMOMETHYLASE2 (CMT2)-dependent DNA methylation pathway. This might relate to the proximity of NPCs to chromocenters in which peri-centromeric long TEs are abundant. Interestingly, in a cross-analysis involving previously determined epigenomic and Hi-C datasets, the authors further found that NPC-proximal chromatin fragments are enriched with hundreds of H3K27me3-marked genes, a feature not reported previously for other species. This finding suggests either a specific recruitment of PRC2 complexes to the nuclear periphery, or the tethering of H3K27me3-marked genes by associated chromatin readers. Collectively, this study reveals that plant NPCs are in close proximity to the two major known repressive chromatin contexts: heterochromatinized TEs and *Polycomb-*repressed genes (Fig. 1b). Determining whether repressive chromatin domains are in direct contact with the NPCs and unraveling the functional significance of this proximity are challenges that remain.

## Perspectives

The study by Bi and colleagues [5] adds to the emerging picture drawn for eukaryotic cells in which the nuclear periphery offers a functional compartment for genome regulation, unveiling antagonistic chromatin contexts in *Arabidopsis*. On the one hand, the association of transcriptionally active chromatin with NPCs can promote efficient mRNA surveillance and export [2] while, on the other hand, the anchoring of large heterochromatic domains and *Polycomb*-repressed silent regions at the nuclear periphery may provide an efficient way to organize or stabilize higher-order chromatin architecture. A potential contribution of H3K27me3-marked domains in tethering long-range euchromatin interactions around the nuclear periphery offers a novel and exciting working hypothesis for further investigations.

A further question that is raised is whether there is a gradient of transcriptionally active chromatin domains from the interior to the periphery in *Arabidopsis* nuclei. PRC2 subnuclear foci are not exclusively enriched at the nuclear periphery, neither is RNA Polymerase II excluded from this area [10], possibly owing to subtle spatial gradients at the nanoscale level that remain to be elucidated. In an effort to provide a unifying model, it has been proposed that NPCs and associated factors may serve as transitional zones between contrasting microenvironments at the nuclear periphery [2, 4]. This model helps to discriminate a counter-intuitive role of NPCs in gene repression from a function in defining physical boundaries preserving transcriptional activity in a globally repressive context, a concept viewed as heterochromatin exclusion [4]. Additional analyses exploiting different components of the nuclear envelope and associated lamina-like matrix, perhaps using methods generating smaller chromatin fragments, are expected to improve our understanding of peripheral chromatin domains, working towards a spatial model with a more refined resolution. Such investigations are required to pinpoint the positional, epigenetic, and transcriptional status of the large gene sets neighboring the nuclear envelope, possibly taking advantage of cell type-specific profiling in combination with in-situ analyses using super-resolution microscopy.

Cell cycle and cellular responses to endogenous and environmental signals trigger dramatic changes in nuclear architecture whose functional impact on genome expression programs remains poorly understood. Future investigations of peripheral chromatin dynamics during cellular transitions thus offer exciting perspectives that can be used to unravel the functional plasticity of plant cell nuclear organization. Exploration of how plant lamina-like components and chromatin-bound factors mediate the anchoring of distinct chromosomal domains in plant species with diverse genomic and chromosomal arrangements should additionally allow the work of Bi and colleagues in *Arabidopsis* to be extended, and uncover the molecular drivers of plant nuclear architecture diversity.

## Abbreviations

*CAB*: *CHLOROPHYLL A/B-BINDING PROTEINS*
NAD: Nucleolus-associated domain
NPC: Nuclear pore complex
NUP1: NUCLEOPORIN1
PRC2: POLYCOMB REPRESSIVE COMPLEX 2
RE-ChIP: Restriction enzyme-ChIP
TE: Transposable element
